# *Mi-1*-Mediated Resistance to *Meloidogyne incognita* in Tomato May Not Rely on Ethylene but Hormone Perception through ETR3 Participates in Limiting Nematode Infection in a Susceptible Host

**DOI:** 10.1371/journal.pone.0063281

**Published:** 2013-05-23

**Authors:** Sophie Mantelin, Kishor K. Bhattarai, Teraneh Z. Jhaveri, Isgouhi Kaloshian

**Affiliations:** 1 Department of Nematology, University of California Riverside, Riverside, California, United States of America; 2 Center for Plant Cell Biology, University of California Riverside, Riverside, California, United States of America; Ghent University, Belgium

## Abstract

Root-knot nematodes, *Meloidogyne* spp., are important pests of tomato (*Solanum lycopersicum*) and resistance to the three most prevalent species of this genus, including *Meloidogyne incognita*, is mediated by the *Mi-1* gene. *Mi-1* encodes a nucleotide binding (NB) leucine-rich repeat (LRR) resistance (R) protein. Ethylene (ET) is required for the resistance mediated by a subset of NB-LRR proteins and its role in *Mi-1*-mediated nematode resistance has not been characterized. Infection of tomato roots with *M. incognita* differentially induces ET biosynthetic genes in both compatible and incompatible interactions. Analyzing the expression of members of the ET biosynthetic gene families *ACC synthase* (*ACS*) and *ACC oxidase* (*ACO*), in both compatible and incompatible interactions, shows differences in amplitude and temporal expression of both *ACS* and *ACO* genes in these two interactions. Since ET can promote both resistance and susceptibility against microbial pathogens in tomato, we investigated the role of ET in *Mi-1*-mediated resistance to *M. incognita* using both genetic and pharmacological approaches. Impairing ET biosynthesis or perception using virus-induced gene silencing (VIGS), the ET-insensitive *Never ripe* (*Nr*) mutant, or 1-methylcyclopropene (MCP) treatment, did not attenuate *Mi-1*-mediated resistance to *M. incognita*. However, *Nr* plants compromised in ET perception showed enhanced susceptibility to *M. incognita* indicating a role for *ETR3* in basal resistance to root-knot nematodes.

## Introduction

Plants have evolved different modes of defense to detect and limit pathogen invasion. Physical damage or mechanical stress caused during the infection process can trigger plant defenses. Alternatively, specific recognition of the invader by the plant host relies on the perception of pathogen associated molecular patterns (PAMPs), signatures that are characteristic of an entire class of pathogens [1], [2]. In plants, this recognition triggers a chain of signaling events that leads to basal defense also known as PAMP-triggered immunity (PTI). To evade PTI, pathogens have evolved effectors that interfere with recognition processes and/or suppress plant defenses. In turn, plants have developed specific recognition factors or resistance (*R*) genes that directly or indirectly detect these effectors and trigger gene-for-gene resistance [3], also known as effector-triggered immunity (ETI; [2]).

Root-knot nematodes (RKN, *Meloidogyne* spp.) are endoparasites that infect large number of crops and cause serious yield losses worldwide [4]. The infective-stage juveniles (J2), hatch from eggs, penetrate behind the root tip and move intercellularly, causing minimum damage, to reach the vascular element where they establish elaborate feeding sites known as giant cells. These specialized cells are multinucleate and provide a source of nutrients for the nematode. In most plant species, giant cells are surrounded by hypertrophied cortical cells forming root knots. Soon after initiation of a feeding site, the J2 becomes sedentary and undergoes three molts to become an adult. Adult females lay eggs in gelatinous matrix or egg masses protruded on the root surface.

In tomato, resistance to three RKN species *M. arenaria*, *M. incognita* and *M. javanica* is conferred by the *Mi-1* gene [5]. *Mi-1*-mediated resistance to RKN in tomato is characterized by a localized hypersensitive response where the nematode attempts to initiate a feeding site [6]. To date, *Mi-1* is the only cloned *R* gene for RKN. In addition to RKN resistance, *Mi-1* confers resistance to potato aphids, whiteflies and tomato psyllids [7], [8], [9].

Gene expression profiling of tomato roots early after *M. incognita* inoculation indicate that RKN differentially regulates all three major plant defense hormones salicylic acid (SA), jasmonic acid (JA), and ethylene (ET) signaling pathways [10]. Although it was previously thought that the SA signaling pathway often contributes to resistance against biotrophic pathogens, while the JA and ET signaling pathways contribute to defense responses against necrotrophic pathogens [11], recent information indicates that all three hormones contribute to defense against both types of pathogens [12].

Roles for SA and JA in tomato defenses against *M. incognita* have been investigated using pharmacological and forward genetic approaches. In a compatible interaction, no effect on nematode reproduction was observed in transgenic *NahG* tomato lines that fail to accumulate SA [10]. Similarly, *Mi-1*-resistance to RKN was not compromised in *Mi NahG* tomato lines, indicating that SA is not essential for the trigger of plant defenses in spite of SA signaling pathway being activated in response to RKN infection. Interestingly, SA is required for the *Mi-1*-mediated resistance to potato aphids in tomato [13]. Alteration of JA perception using the *jai1-1* (*jasmonic acid insensitive 1*) mutation in tomato did not impair *Mi-1*-mediated resistance to RKN [14]. However, the *jai1* mutant displayed reduced susceptibility to RKN in a compatible host indicating that tomato susceptibility to RKN requires an intact JA signaling pathway. Taken together, these results highlight the diverse mode of actions in *Mi-1* resistance.

In tomato, ET has been associated with both induction of host defense responses [15], [16] as well as promoting pathogen virulence and disease [17], [18], [19]. ET production during pathogen infection is mostly controlled at the transcriptional level, through regulation of genes encoding ACC synthase (ACS) and ACC oxidase (ACO) which catalyze the two committed steps of ET biosynthesis [20]. Both ACS and ACO are encoded by multigene families and members of these families are transcriptionally regulated differently during development and under distinct stress conditions. Perception of ET is also an important factor in regulating ET signaling. Tomato has six ET receptors (*ETR1-6*) and each has a distinct pattern of expression throughout development and in response to external stimuli [21]. *ETR3* (also known as *never ripe* (*nr*)) and *ETR4* in particular, appear to function as negative regulators of the ET signaling pathway in the absence of the hormone and are known to be induced by pathogen infection [22], [23], [24]. They are also inducible by ET itself, a feedback loop of regulation which may serve to regulate the magnitude and duration of ET responses [23], [25], [26].

In this study, we demonstrate that an increase in expression of ET biosynthetic genes occurs early in tomato roots in both compatible and incompatible interactions with *M. incognita*. To functionally assess the role of ET in *Mi-1-*mediated RKN defense, we concurrently used genetic and pharmacological approaches to impair ET perception in susceptible and *Mi-1*-resistant tomato plants. In addition, we targeted genes involved in ET biosynthesis for silencing in resistant *Mi-1* containing plants. Our results demonstrated a role for the ET receptor ETR3 in limiting RKN infection in compatible interaction however no essential role for ET was identified in *Mi-1*-mendiated RKN resistance.

## Results

### ET signaling in tomato roots is activated during the early stages of RKN infection

In a previous study [10], microarray analysis identified a large set of genes regulated in tomato roots in both resistant cv. Motelle (*Mi-1/Mi-1*) and susceptible cv. Moneymaker (*mi/mi*) plants 24 h after *M. incognita* infection. About 1.3% of the corresponding probes on the array (TOM1 tomato array) are ET-related genes (Table S1). These correspond to 21 probes representing 16 different genes belonging to three classes of ET-related genes: ET receptor, ET biosynthetic and ET responsive genes. Most of these genes are differentially up-regulated (*P*<0.05) in tomato roots by RKN infection. Interestingly, among the three ET receptors, *ETR1*, *ETR2* and *ETR3,* represented on the array only *ETR3* was significantly up-regulated upon RKN infection (Table S1). In addition at least 3 *ACS* genes, *ACS1A*, *ACS2*, and *ACS6,* were up-regulated (Table S1).

**Figure 1:**
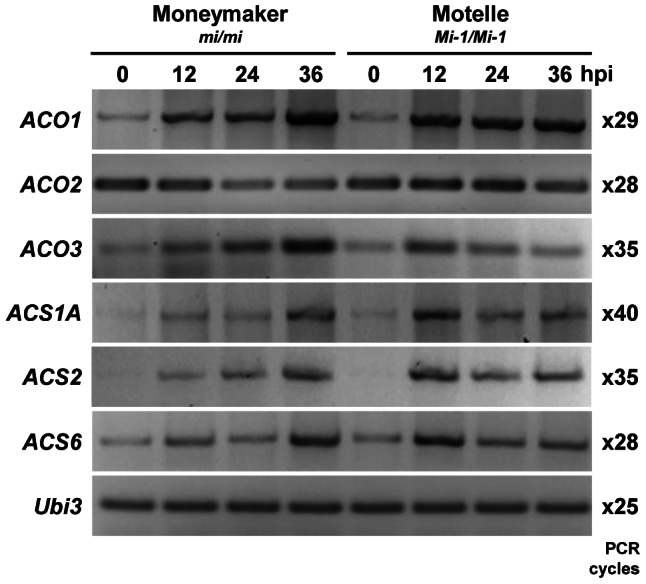
Root-knot nematodes (*Meloidogyne incognita*) induce the expression of ethylene biosynthetic genes in tomato. *In vitro* grown seedlings of near isogenic tomato cvs. Moneymaker and Motelle were infected with 100–150 second-stage juvenile root-knot-nematodes in sterile conditions. The infected root tips were sampled at 0, 12, 24 and 36 h post infection (hpi). Expression of 1-aminocyclopropane-1-carboxylic acid (ACC) oxidase genes (*ACO*) and ACC synthase genes (*ACS*) was determined by semi-quantitative RT-PCR using gene-specific primers (Table S1) in two biological replicates with similar results. PCR amplification from a single sample is presented for each time point and genotype. Amplification of the tomato ubiquitin *Ubi3* gene was used as internal control. PCR cycles are indicated on the right side of the panel.

ET biosynthesis is controlled by the modulation of both ACS and ACO activities and transcriptional regulation of *ACS* and *ACO* gene family members [27]. To confirm the involvement of ET in response to RKN in tomato, we examined ET biosynthetic genes, by monitoring the temporal expression of three *ACO* genes, *ACO1*, *ACO2* and *ACO3*, and three *ACS* genes, *ACS1A*, *ACS2*, and *ASC6* using semi-quantitative reverse transcription-PCR (RT-PCR) in tomato roots of susceptible cv. Moneymaker and resistant cv. Motelle after RKN inoculation (Table S2). *ACO2* was constitutively expressed while transcripts of all other tested *ACO* and *ACS* genes were weakly expressed or non-detectable in un-inoculated roots of both tomato cultivars (Figure 1). *ACO1* transcripts accumulated in both tomato cultivars at 12 h post inoculation (hpi) and transcript abundance remained high throughout the experiment. *ACO1* transcript levels peaked faster in cv. Motelle (12 hpi) compared to cv. Moneymaker (36 hpi). *ACO3* transcripts were not as abundant as *ACO1* and although *ACO3* also peaked faster in cv. Motelle (12 hpi) compared to cv. Moneymaker (36 hpi), *ACO3* transcript levels decreased soon after the peak in cv. Motelle (Figure 1). By contrast, expression of *ACO2* decreased after RKN inoculation in both susceptible and resistant plants, although at faster pace in susceptible roots (Figure 1). RKN inoculation induced the expression of all three *ACS* genes tested in both susceptible and resistant plants. In both tomato cultivars, the temporal expression of *ACS1A*, *ACS2* and *ACS6* were similar to that of *ACO3* gene, with transcript levels peaking faster in cv. Motelle (12 hpi) compared to cv. Moneymaker (36 hpi) and decreasing soon after in cv. Motelle (Figure 1).

### Compromising the ET biosynthetic pathway does not affect *Mi-1* resistance to RKN

RKN inoculation regulated the expression of ET biosynthetic genes in tomato roots. Since the temporal pattern was markedly different in resistant compared to susceptible tomato, we tested whether silencing *ACS* genes will attenuate *Mi-1-*mediated resistance to RKN. The ACS enzyme catalyzes the first committed step and in most cases is the rate-limiting step in ET biosynthesis [28]. Two tobacco rattle virus (TRV)-based constructs, TRV-ACSI and TRV-ACSII, were used in virus-induced gene silencing that should enable silencing of six *ACS* genes when combined (Table S3; [29]). These two constructs were agroinfiltrated alone or combined into cv. Motelle leaves for RKN infection assays.

These two TRV-ACS constructs were tested previously, individually and in combination, for their gene silencing specificity and efficiency in tomato leaves [29]. To evaluate *ACS* genes silencing in TRV agroinfiltrated plants infected with RKN, we evaluated the effect of the combined TRV-ACSI+II constructs on the expression of the six-targeted *ACS* genes in roots using quantitative RT-PCR. The combined constructs were able to silence *ACS1B*, *ACS2* and *ACS6* albeit at variable levels (Figure S1). *ACS1A*, *ACS4* and *ACS5* transcripts could not be detected in tomato roots irrespective of silencing (data not shown). These three *ACS* genes could be amplified from genomic DNA using the same pair of primers (data not shown), indicating that they are not expressed at detectable levels in tomato roots under our growth conditions. The efficiency of TRV-ACSI+II constructs to silence *ACS1A* has been demonstrated previously in leaves [29]. Although at very low levels, *ACS1A* transcripts could be detected in roots after *in vitro* RKN infection of tomato root tips (Figure 1, Table S1). The inability to detect *ACS1A* transcripts in the TRV-treated plants could be due to the very different plant growth conditions (potted plants vs. root tips), or RKN infection method and timing or both.

**Figure 2:**
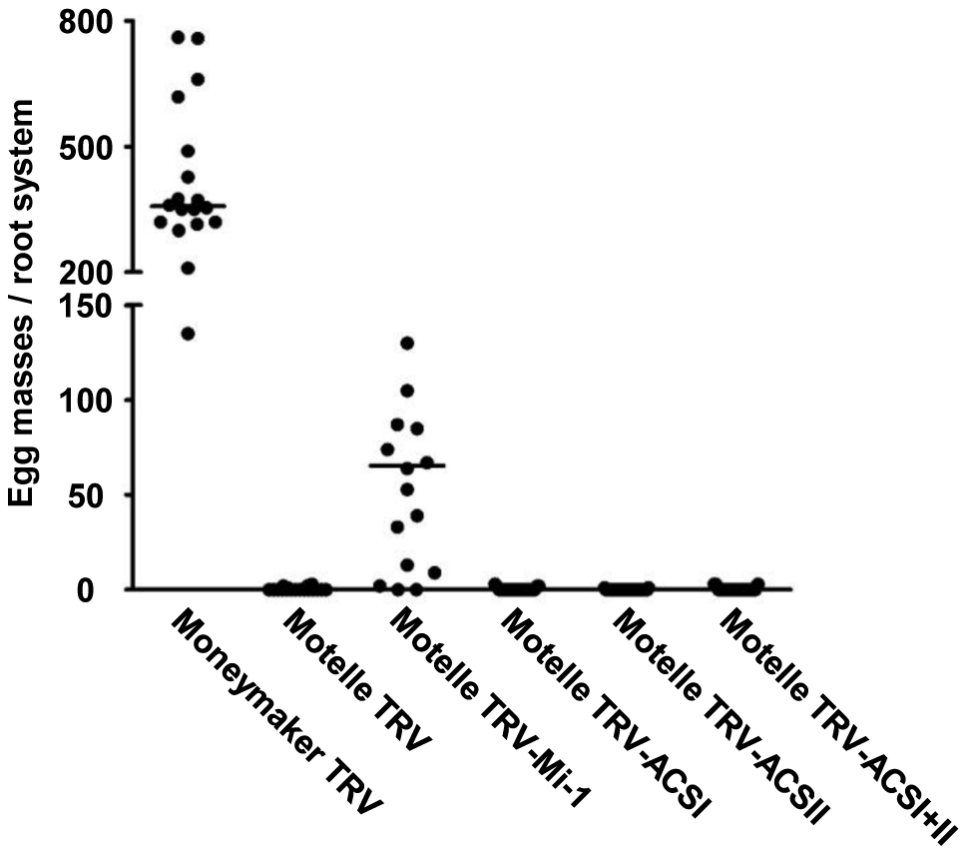
Silencing *ACS* genes in tomato does not compromise *Mi-1*-mediated resistance to root-knot nematodes. Two-week-old tomato plants cvs. Moneymaker (*mi/mi*) and Motelle (*Mi-1/Mi-1*) were used in agroinfiltration of tobacco rattle virus (TRV) empty vector, and cv. Motelle was used with TRV containing a portion of *Mi-1* (TRV-Mi-1) or containing *1-aminocyclopropane-1-carboxylic acid synthase* (*ACS*) constructs (TRV-ACSI and TRV-ACSII), which were either individually or simultaneously agroinfiltrated (TRV-ACSI+II). Three weeks after agroinfiltration, plants were infected with 10,000 second-stage juvenile root-knot-nematodes and evaluated for nematodes reproduction 8 weeks later. Dots represent the number of egg masses counted on a single root system (*n* = 18–25). Two independent experiments were performed with similar results and data from one are presented.

Evaluation of RKN infection, by counting the number of egg masses per root system, indicated that none of the TRV constructs, alone (TRV-ACSI or TRV-ACSII) or in combination (TRV-ACSI+II), were able to attenuate *Mi-1*-mediated resistance in cv. Motelle tomato (Figure 2). In this same experiment, RKN were able to infect roots of cv. Motelle agroinfiltrated with a TRV construct targeting the *Mi-1* gene (TRV-Mi-1) but not the TRV-infected control plants, indicating that we were able to silence a gene in roots and attenuate *Mi-1*-mediated resistance using this approach (Figure 2). However, RKN infection in cv. Motelle TRV-Mi-1 roots was variable and lower than on cv. Moneymaker confirming previous observation that silencing in roots is partial and not uniform [30].

### Blocking ET perception in roots using MCP

**Figure 3:**
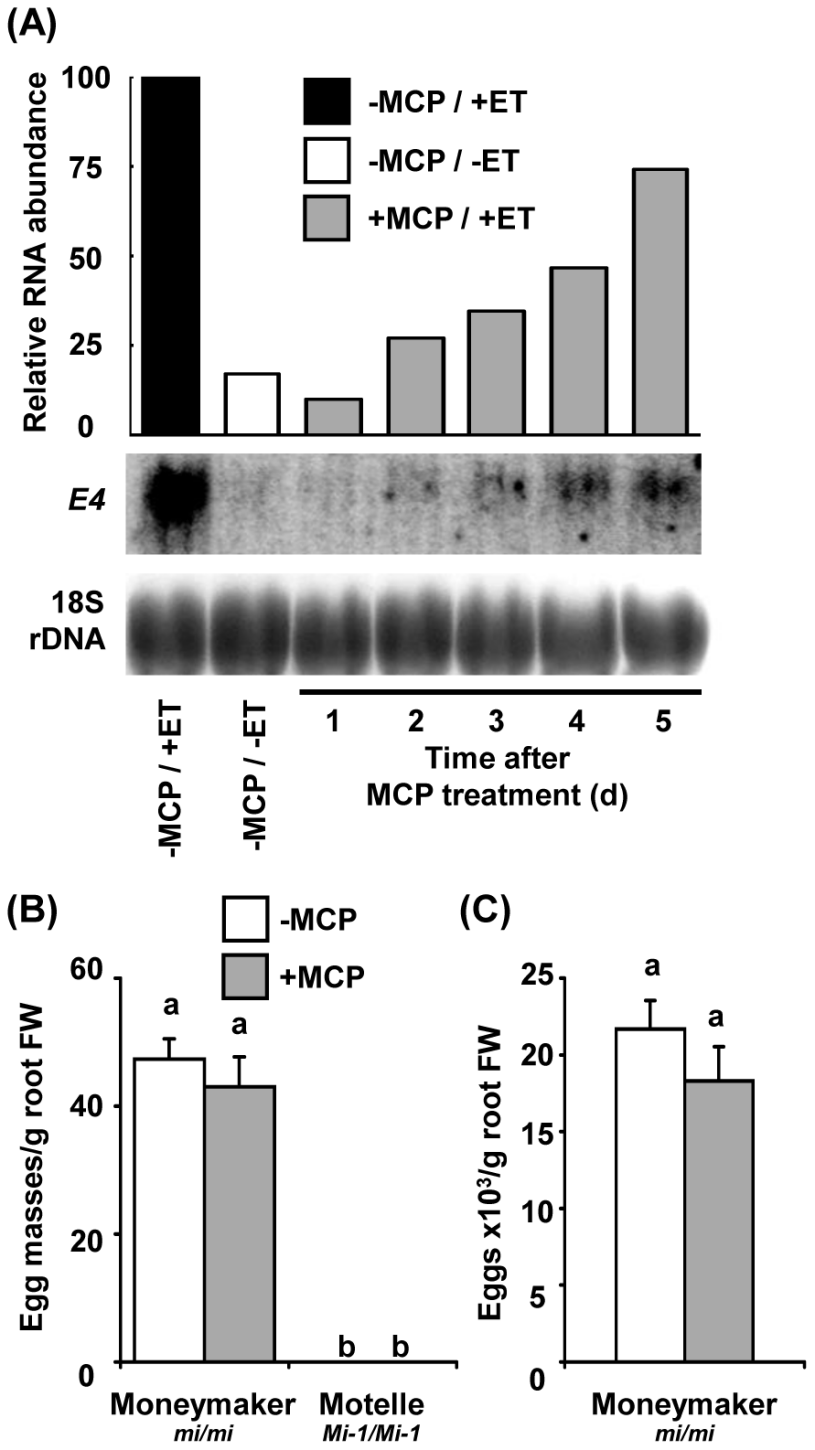
Effect of MCP treatment on ethylene response and resistance to root-knot nematode in tomato roots. (**A**) Efficiency of the 1-methylcyclopropene (MCP)-blocking of ethylene (ET) perception was assessed by monitoring the expression of *E4* after induction by ET. Seven-week-old cv. Moneymaker plants (+MCP/+ET) were pre-treated with MCP, and two plants were treated daily for 18 h with 10 µl/l ET prior to harvest. Root tissues were pooled and frozen. Tissues from untreated plants (−MCP/−ET) or plants only induced by ET (−MCP/+ET) were used as control. Total RNA (25 µg) for each sample was used for RNA blot analysis. The blot was hybridized sequentially with *E4* and an 18S rDNA probe used to normalize expression. (**B**, **C**) Five-week-old tomato plants cvs. Moneymaker and Motelle were treated with MCP (+MCP) or untreated (−MCP) prior root-knot nematode (RKN) infection with 3,000 second-stage juvenile. During the first 2 weeks following RKN infection, the plants (+MCP) were repeatedly treated with MCP every 2 days. RKN reproduction was evaluated 7 weeks after infection as (**B**) egg masses and (**C**) egg production. Results are presented relative to the fresh weight (FW) of roots. Error bars indicate standard error of the mean (*n* = 16), where bars with different letters denote significant difference at *P*<0.05. The bioassay was performed twice with both tomato cultivars tested and twice more with cv. Moneymaker only. In all experiments, results from the same genotypes were similar. Data from one representative experiment are presented.

We implemented a second approach to evaluate the contribution of ET in *Mi-1*-mediated RKN resistance by impairing ET perception using 1-methylcyclopropene (MCP). MCP functions as a competitive inhibitor of ET and its attachment to the receptors is essentially irreversible [31]. The use of MCP to block ET perception in roots has not been evaluated previously. Thus, we first assessed the ability of MCP to block ET receptors over time in tomato roots. Expression of the ET-inducible gene *E4* was examined in roots of tomato cv. Moneymaker treated with MCP and subsequently induced with ET 1 to 5 days later. Pre-treatment of tomato with MCP decreased basal expression of *E4* and prevented ET-induced *E4* transcript accumulation for one day (Figure 3A), indicating that ET perception in tomato roots was successfully blocked. However, two days after MCP treatment, about 27% of the *E4* induction was recovered and this continued to increase over the rest of the five-day period analyzed. In order to maintain strong blockage of ET perception, plants were required to be treated frequently with MCP during RKN infection, establishment of a feeding site and nematode development. Therefore, the effect of MCP on RKN infectivity was assessed. RKN J2 were treated with the same concentration of MCP as that for plants and used for inoculation of susceptible tomato cv. Moneymaker. Untreated nematodes were used as control. Six weeks after inoculation, no difference in number of egg masses produced by treated and untreated J2 (47±4 and 49±3 egg masses/g of fresh root weight in non-treated and MCP-treated J2, respectively; average ± SE for *n* = 20) were observed indicating that MCP did not affect RKN infectivity.

Resistant cv. Motelle and susceptible cv. Moneymaker plants were treated with MCP, inoculated with J2, and repeatedly treated with MCP every two days during a period of 2 weeks. Six weeks after inoculation, no egg masses were observed on cv. Motelle roots treated or untreated with MCP (Figure 3B). As in the previous experiment, RKN was able to infect and reproduce on cv. Moneymaker roots with no significant differences (*P*<0.05) between MCP treated and untreated plants (Figure 3B and 3C).

### *ETR3*-mediated ET signaling is not required for *Mi-1* resistance but contributes to basal resistance in the compatible host

Although MCP treatment reduced ET perception in plants it did not compromise *Mi-1*-mediated resistance to RKN or affect the susceptibility of the compatible host. However, the effect of MCP is not permanent and low levels of ET perception in MCP-treated roots could be sufficient for RKN resistance. Therefore, we used the only available ET receptor mutant *Never ripe* (*Nr*), which is ET-insensitive [32], [33]. *Nr* is a co-dominant mutation that arose from a single base substitution in the N-terminal coding region of the tomato *ETR3* gene and has been introduced into the *Mi-1* genetic background [25], [29]. The characteristic ET growth-inhibiting effect is attenuated in this *Mi-1 Nr* line similar to the *Nr* mutant line [29], [32].

**Figure 4:**
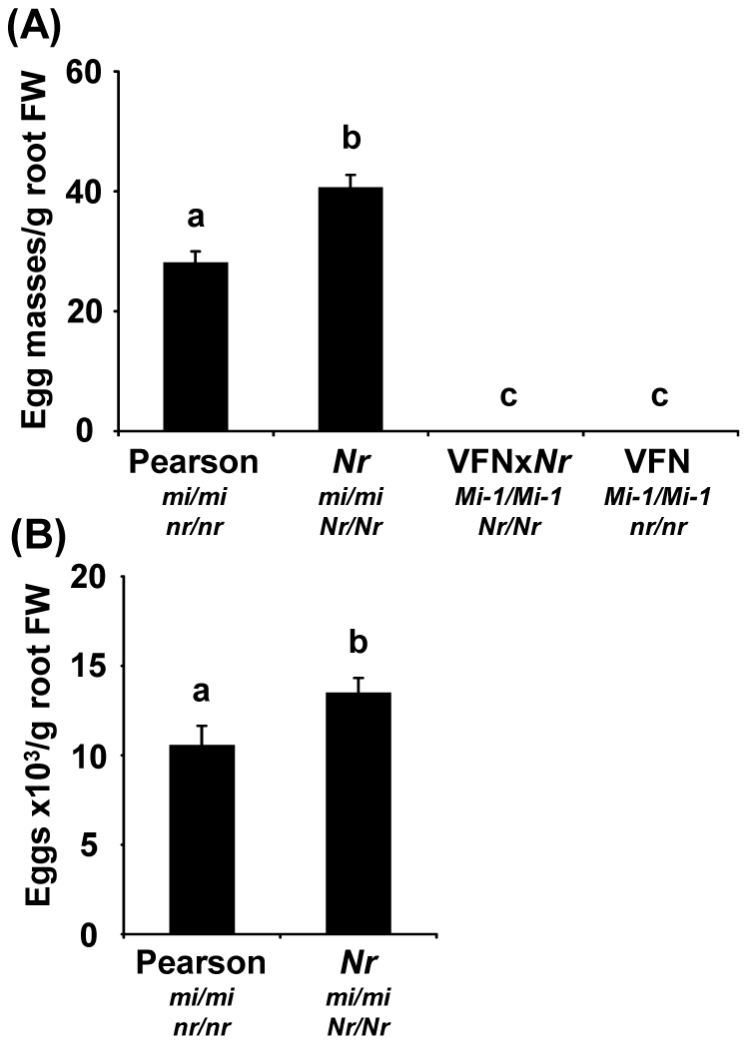
Root-knot nematodes reproduction on tomato is affected by the *Nr* mutation in compatible host only. Root-knot nematodes (RKN) reproduction was evaluated on *Never ripe (Nr)* mutant, wild type tomato cvs. Pearson and VFN, and the *Nr* introgressed line VFNx*Nr*. Four-week-old plants were infected with 3,000 second-stage juvenile RKN. (**A**) Egg masses and (**B**) eggs production were evaluated 6 weeks after RKN infection. Results are presented relative to the fresh weight (FW) of roots. Error bars indicate standard error of the mean (*n* = 20–30), where bars with different letters denote significant difference at *P*<0.05. Two independent experiments were performed with similar results and data from one are presented.

Homozygous *Mi-1 Nr* plants, parental susceptible lines *Nr* and the wild-type cv. Pearson as well as resistant parent VFN were evaluated for RKN infection. No egg masses were observed on VFN plants irrespective of the presence of the *Nr* mutation (Figure 4A). In contrast, the number of egg masses on *Nr* plants was significantly higher than on the wild-type parent cv. Pearson (Figure 4A). Similarly, the number of eggs per gram of root was also significantly higher on *Nr* plants compared to wild-type parent cv. Pearson suggesting that *ETR3* is involved in basal resistance against RKN but is not required for *Mi-1-*mediated resistance (Figure 4B).

## Discussion

In compatible interactions, nematodes must cope with the plant's constitutive and inducible defenses in order to establish and maintain their feeding structure. But there are no consensual roles for ET in response to nematodes. Each interaction seems to play by its own rules, that partly relates with the mode of interaction between the plant and the parasite. Analysis of ET-insensitive plants has demonstrated a role for ET in plant-nematode compatible interactions, yet the effect of ET on nematode virulence varies greatly. ET insensitivity reduces root colonization by the soybean cyst nematode *Heterodera glycines* [34]. Similarly, Arabidopsis mutants with reduced ET sensitivity are less susceptible to infection by the sugar beet cyst nematode *H. schachtii* but display enhanced susceptibility to the RKN *M. hapla* [35], [36]. In rice, ET insensitivity also leads to enhanced susceptibility to the RKN *M. graminicola* [37]. ET probably has pleiotropic roles in plant-nematode interactions. In the early stages of nematode infection, ET may be involved in plant defense signaling, while it later stages acts as a growth regulator mediating the formation and expansion of the syncytia induced by cyst nematodes or enhancing the expansion of cortical parenchyma cells leading to gall formation induced by RKN infection and allowing expansion of the giant cells by inhibiting the lignification of the surrounding cells [36], [38].

ET synthesis increases during RKN *M. javanica* infection in tomato [38]. In agreement to this finding, we demonstrated that in the early stages of *M. incognita* infection of tomato roots, the ET biosynthesis pathway is transiently activated. A detailed time course of *ACO* and *ACS* gene expression in tomato roots infected with RKN showed increases in most transcript levels as early as 12 h after inoculation in both susceptible and resistant roots. However, differences in the magnitude and the temporal expression were detected between the two genotypes. The delay in *ACO/ACS* transcripts accumulation in response to RKN in the susceptible host compared to *Mi-1* resistant plants rather suggest a differential control of RKN-induced ET biosynthesis in the two genotypes which may translate to a difference in plant resistance response. Defense genes are typically activated faster and to higher magnitude in resistant plants compared to susceptible plants [39], [40]. We therefore tested whether ET biosynthesis is required for *Mi-1* mediated resistance.

In response to RKN, tomato plants contain higher levels of the ET precursor ACC indicating that nematode infection induces an increase in ACS activity [41]. We performed RKN bioassays using resistant cv. Motelle plants silenced for multiple *ACS* genes, including *ACS1A*, *ACS2* and *ACS6* which are induced by RKN infection. However, we found no effect of the silencing on *Mi-1*-mediated RKN resistance suggesting that the differences in gene expression did not translate to resistance. Although lowering *ACS* genes expression using VIGS had no impact on *Mi-1*-mediated resistance, we can't exclude the possibility that the decrease in transcript levels was not sufficient to affect ET biosynthesis and thus definitively exclude a role for this hormone in *Mi-1* resistance to RKN.

Modulation of ET action can also occur by changes in ET sensitivity which is mediated by hormone receptors. Tomato perceives ET with at least six putative receptors (ETRs), and the ET signal is then transmitted to a family of downstream kinases (CTRs). In the absence of ET, ETRs act as negative regulators as they activate CTRs to suppress the downstream ET response, while ET binding deactivates the receptors and switches on downstream signalling events [23], [42]. Blocking ETRs using ET competitors should then render the plants less sensitive to ET.

In this study, tomato plants were treated with MCP which irreversibly binds to ET receptors and consequently blocks ET-mediated signalling. The pharmacological treatment successfully prevented the ET-dependent transcriptional activation of *E4* in the tomato roots, indicating loss of ET sensitivity. However, blocking ET perception using MCP did not affect *Mi-1*-mediated resistance or modify susceptibility to RKN in a compatible interaction. Partial ET sensing is recovered shortly after MCP treatment suggesting a rapid turnover of the receptors in root cells. In tomato immature fruits and vegetative tissues, multiple ET receptors including ETR3 are degraded in response to ET treatment despite increases in the receptor gene transcript levels [26]. Degradation of ETRs through the proteasome is due to the ligand-receptor binding, inducing the turnover of the receptors, and not due to downstream ET responses [26]. A similar process seems to be triggered by MCP binding to ET receptors inducing the turnover of the proteins in roots.

To directly assess a role for ET sensitivity in response to RKN, we used the only available ET receptor mutant, *Nr,* that has been implicated in response to pathogens [17], [19], [23]. The *Nr* mutation confers ET insensitivity in tomato [32]. The introgression of the *Nr* mutation into a *Mi-1* background did not compromise resistance to RKN suggesting that ET sensitivity in tomato is dispensable for *Mi-1*-mediated resistance to RKN. However, the *Nr* mutant is known to retain a residual ET response [43] and a minimum threshold of ET sensing may be sufficient to achieve optimum RKN defense. Based on tomato response to *Xanthomonas campestris* pv. *vesicatoria*, it has been suggested that induction of the *ETR* genes during an incompatible interaction limits cell death at the site of infection by decreasing the ET sensitivity of the surrounding tissue [23]. In *Mi-1*-resistant tomato roots, RKN infection also triggers a typical hypersensitive response [6] and the increase in *ETR3* transcript levels specifically in resistant roots may be related to a similar function.

Although the *Nr* mutation in the *Mi-1* background did not affect tomato resistance to RKN it enhanced RKN susceptibility in compatible plants. The higher RKN infection rate observed in *Nr* mutant compared to its wild-type parent cv. Pearson is consistent with the recent observation that infective juveniles of *M. hapla* are more attracted to *Nr* mutant roots than to wild-type tomato [35]. Since similar basal levels of ET is produced in *Nr* and wild-type plants and pathogen-induced ET production is not compromised in *Nr* plants [19], [23], ET-dependent signaling and not ET production modulates attractiveness of tomato roots to RKN. The enhanced susceptibility of the *Nr* mutant to RKN could therefore be partially attributed to the modulation of RKN attraction to roots. Enhanced aphid attraction to *Nr* plants compared to wild-type tomato was also observed, although this attraction did not result in enhanced colonization by the insect [29]. Since *Nr* plants are impaired in regulation of ET-inducible genes, *ACO3* in particular [15], the differential transcriptional regulation of *ACO* genes by RKN infection suggests that *Nr* mutant might be affected in RKN-mediated ET synthesis, preventing the establishment of an optimum basal resistance. Taken together, enhanced susceptibility of *Nr* plants to RKN could be contributed by enhanced attractiveness of roots and impaired resistance through ETR3.

## Materials and Methods

### Plant material and growth conditions

Tomato (*Solanum lycopersicum*) lines used in this study were: near isogenic cvs. Motelle (*Mi-1*/*Mi-1*) and Moneymaker (*mi*/*mi*), cv. VFN (*Mi-1*/*Mi-1*), the *Never ripe* (*Nr*) mutant (*mi*/*mi Nr*/*Nr*) and its wild-type parent cv. Pearson (*mi*/*mi nr*/*nr*). Unless otherwise stated, seed were treated with 10% (vol/vol) bleach and germinated in seedling trays in organic planting mix (Sun-Gro Horticulture, Bellevue, WA, USA) supplemented with Osmocote (17-6-10; Sierra Chemical Company, CA, USA), and maintained in a mist room. Two weeks after germination, seedlings were transplanted into pots (10 cm in diameter and 17 cm deep) filled with UC mix containing sand and organic matter (90/10 vol/vol) supplemented with Osmocote. Plants were grown in a greenhouse with temperatures 22 to 26°C and fertilized bimonthly with MiracleGro (Stern's MiracleGro Products, Port Washington, NY, USA).

After transplanting, plants used for VIGS experiments were grown in growth chambers at 19°C until nematode inoculation. Detailed plant growth conditions for VIGS experiments were described previously [30]. Briefly, 2 weeks after transplanting, seedlings with a pair of newly emerged leaves were agroinfiltrated with TRV constructs. Two to three weeks later, when the TRV-PDS treated plants showed photobleached leaf symptoms, plants were inoculated with nematodes and maintained at 24°C in a growth chamber. Two weeks later, plants were moved to a greenhouse and maintained at 22 to 26°C until evaluation.

For *in vitro* RKN infection, tomato seeds were surface sterilized in 10% (vol/vol) bleach and germinated in sterile conditions on Whatman paper in the dark as described by Lambert and associates [44].

### Genetic crosses and homozygous (*Mi-1 Nr*) plant selection

Genetic crosses between cv. VFN (*Mi-1*/*Mi-1 nr*/*nr*) and the *Nr* mutant (*mi*/*mi Nr*/*Nr*) and selection of plants homozygous for *Mi-1* and the *Nr* mutation (*Mi-1/Mi-1 Nr/Nr*) were described previously [29]. Bulked seeds from selfed F3 populations, homozygous for *Mi-1* and the *Nr* mutation, were used.

### Constructs used for virus-induced gene silencing

We used tobacco rattle virus (TRV)-based VIGS to repress candidate genes. The TRV-VIGS constructs TRV-ACSI and TRV-ACSII used to silence the tomato *ACS* genes were described previously (Table S3; [29]). We also used as control the previously described TRV-Mi and TRV-PDS constructs to silence the tomato *Mi-1* and phytoene desaturase *PDS* genes, respectively [13], [45]. All TRV-VIGS clones were transformed into *Agrobacterium tumefaciens* strain GV3101.

### *Agrobacterium tumefaciens*-mediated virus infection

Cultures of *A. tumefaciens* strain GV3101 containing each of the constructs, empty vector pTRV2, or pTRV1 [45] were grown and prepared as previously described [13], [29]. Bacteria were resuspended in infiltration buffer at OD_600_ 1.0. Cells were incubated at room temperature for 3 h before use. Equal volumes of *A. tumefaciens* pTRV1 and pTRV2 were mixed and used for infiltration (agroinfiltration) of leaflets of two to three-week-old seedlings using a 1-ml syringe.

### MCP and ethylene treatments

SmartFresh (0.14% 1-methylcyclopropene [MCP]) was obtained from AgroFresh Inc. (Philadelphia, PA, USA). Tomato plants were treated for 24 h with MCP released to a final concentration of about 0.1 µl/l in an airtight container as described previously [29]. For ET treatment, tomato plants were placed in the airtight container and exposed to 10 µl/l ET gas (California Tool & Welding Supply Company, Riverside, CA, USA) for 18 h as described previously [29]. Potassium hydroxide was included in the container to prevent carbon dioxide accumulation during both MCP and ET treatments [46]. Untreated control plants were held in air and treated plants were aerated for two hours before nematode inoculation.

### RNA blot analyses

Total RNA was isolated using hot phenol, and subjected to RNA gel blot analyses as described previously [14]. The tomato EST clone cTOA29O3 was used to probe for *E4* (gene locus Solyc03g111720) and 18S rDNA probe was used as control to ensure equal loading and transfer. Probes were labeled with ^32^P-α-dCTP, using the Prime-A-Gene labeling kit (Promega). Hybridization was carried out overnight at 42°C in 50% (v/v) formamide, and the final wash was at 65°C in 0.5× SSC, 0.1% SDS (w/v).

### Semi-quantitative reverse transcription-polymerase chain reaction (RT-PCR)

Total RNA was isolated using hot phenol [47]. Twenty micrograms of total RNA were treated with the RQ1 RNase-free DNase (Promega) followed by phenol/chloroform extraction. First strand cDNAs were synthesized from 5 µg DNase-treated RNA using Super-Script II reverse transcriptase (Invitrogen). For PCR, the different transcripts were amplified (94°C for 3 min, cycled [94°C for 45 s, annealing (Table S2) for 30 s, and 72°C for 1 min], and 72°C for 5 min) from 1 µl cDNA in 25 µl reaction using gene-specific primers (Table S2). The tomato ubiquitin *Ubi3* gene was used as a control. To check for the absence of genomic DNA contamination, 200 ng of DNase-treated RNA was used as template.

### Nematode culture and bioassays

The *Mi-1*-avirulent *M. incognita* isolate P77R3 was maintained on susceptible tomato cv. UC82B in a greenhouse. RKN eggs and J2 were obtained from infected roots as described earlier [48]. J2 were collected every 48 h and used immediately. For VIGS experiments, three weeks after agroinfiltration, plants were inoculated with 10,000 J2. In each experiment, 18 to 25 cv. Motelle plants per construct were infected. In addition, 18 cv. Moneymaker plants were agroinfiltrated only with the empty TRV vector control and used as control for nematode virulence. For the MCP and *Nr* screens, four week-old tomato plants were inoculated with 3,000 J2. Inoculated plants were maintained at 22°C to 26°C. For all assays, nematode reproduction was evaluated six to eight weeks after inoculation by staining roots in 0.001% erioglaucine (Sigma) and counting the egg masses on individual root system and/or extracting and counting eggs.

*In vitro* RKN infection was carried out aseptically as described by Lambert and associates [44]. Briefly, *in vitro* grown seedlings with approximately 1.5-cm root length were inoculated with 100 to 150 J2 in sterile 0.5% (wt/vol) carboxymethyl cellulose (Sigma). Control seedlings were inoculated with the same volume of 0.5% (wt/vol) carboxymethyl cellulose. Infected root tips were sampled at 0, 12, 24 and 36 h post infection (hpi), quickly frozen and stored at –80°C.

### Statistical analyses

Statistical analysis for each experiment was performed separately. Analysis of variance (ANOVA) was performed using the STATISTICA 6.0 software (Statsoft, Maisons-Alfort, France) and significant differences between means were evaluated using the Tukey HSD test. Results from replicated bioassays gave similar trends at the same *P* value.

## Supporting Information

Figure S1 **Evaluation of** ***ACS*** **genes silencing in tomato roots.** Expression of 1-aminocyclopropane-1-carboxylic acid (ACC) synthase genes (*ACS*) was determined by quantitative RT-PCR (qRT-PCR) using gene-specific primers (Table S2) in two cv. Motelle roots [samples (1) and (2)] co-agroinfiltrated with TRV-ACSI+II or empty vector TRV. Values represent the means ± SE of three technical replicates normalized relative to tomato ubiquitin *Ubi3* gene. Three weeks after co-agroinfiltration of two silencing constructs TRV-ACSI+II, or empty vector TRV- control, tomato plants were inoculated with 10,000 second-stage juveniles of *Meloidogyne incognita*. Three days after nematode inoculation, a portion of the roots was collected from individual plants for gene expression analysis. Silencing efficiency of A*CS1A*, *ACS1B*, *ACS2*, *ACS4*, *ACS5* and *ACS6* was evaluated in these root samples by qRT-PCR. Transcripts of *ACS1A*, *ACS4* and *ACS5* could not be detected in the control tomato roots. Therefore, results for only *ACS1B*, *ACS2* and *ACS6* are presented. For qRT-PCR, transcripts were amplified from 1 µl of 5× diluted cDNA in a 15 µl reaction using gene-specific primers (Table S2) and iQ^TM^ SYBR Green Supermix (Bio-Rad) following the protocol: 94°C for 5 min, cycled 45× [94°C for 30 sec, 58°C C for 30 sec, and 72°C for 30 sec], and 72°C for 3 min, followed by generation of a dissociation curve. The generated threshold cycle (Ct) was used to calculate the transcript abundance relative to the housekeeping genes (tomato *Ubi3*) as described by Ginzinger (2002) [49].
(PPT)

Table S1 **List of ethylene-related differentially expressed genes in tomato upon root-knot nematode infection (subset of data published in Bhattarai** ***et al.*** **, 2008).**
(XLSX)

Table S2 **Primers used for gene expression analyses.**
(XLS)

Table S3 **Virus-induced gene silencing constructs and their relative silencing efficiency in tomato when used in combination.**
(XLSX)
