# Supplementary material for: Mi-1-Mediated Resistance to Meloidogyne incognita in Tomato May Not Rely on Ethylene but Hormone Perception through ETR3 Participates in Limiting Nematode Infection in a Susceptible Host
Source: PLoS One. 2013 May 23;8(5):e63281. doi: 10.1371/journal.pone.0063281 (PMC3662669; doi:10.1371/journal.pone.0063281)
Supplement: Figure S1 — Evaluation of ACS genes silencing in tomato roots. Expression of 1-aminocyclopropane-1-carboxylic acid (ACC) synthase genes (ACS) was determined by quantitative RT-PCR (qRT-PCR) using gene-specific primers (Table S2) in two cv. Motelle roots [samples (1) and (2)] co-agroinfiltrated with TRV-ACSI+II or empty vector TRV. Values represent the means ± SE of three technical replicates normalized relative to tomato ubiquitin Ubi3 gene. Three weeks after co-agroinfiltration of two silencing constructs TRV-ACSI+II, or empty vector TRV- control, tomato plants were inoculated with 10,000 second-stage juveniles of Meloidogyne incognita. Three days after nematode inoculation, a portion of the roots was collected from individual plants for gene expression analysis. Silencing efficiency of ACS1A, ACS1B, ACS2, ACS4, ACS5 and ACS6 was evaluated in these root samples by qRT-PCR. Transcripts of ACS1A, ACS4 and ACS5 could not be detected in the control tomato roots. Therefore, results for only ACS1B, ACS2 and ACS6 are presented. For qRT-PCR, transcripts were amplified from 1 µl of 5× diluted cDNA in a 15 µl reaction using gene-specific primers (Table S2) and iQTM SYBR Green Supermix (Bio-Rad) following the protocol: 94°C for 5 min, cycled 45× [94°C for 30 sec, 58°C C for 30 sec, and 72°C for 30 sec], and 72°C for 3 min, followed by generation of a dissociation curve. The generated threshold cycle (Ct) was used to calculate the transcript abundance relative to the housekeeping genes (tomato Ubi3) as described by Ginzinger (2002) [49]. (PPT) [file pone.0063281.s001.ppt]

## Slide 1
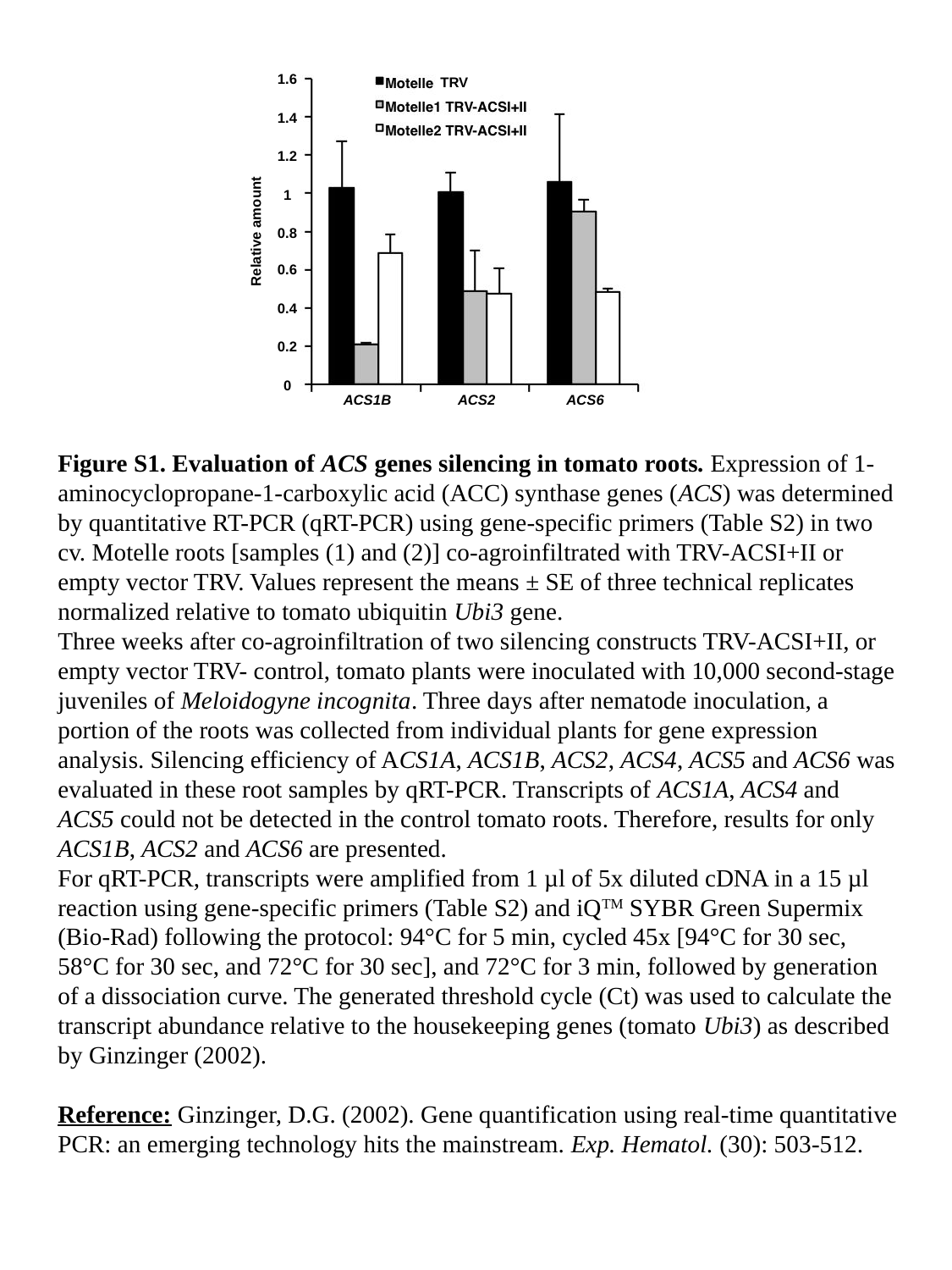

1.6
1.4
1.2
Relative amount
1
0.8
0.6
0.4
0.2
0
ACS1B
ACS2
ACS6
TRV
Figure S1. Evaluation of ACS genes silencing in tomato roots. Expression of 1-aminocyclopropane-1-carboxylic acid (ACC) synthase genes (ACS) was determined by quantitative RT-PCR (qRT-PCR) using gene-specific primers (Table S2) in two cv. Motelle roots [samples (1) and (2)] co-agroinfiltrated with TRV-ACSI+II or empty vector TRV. Values represent the means ± SE of three technical replicates normalized relative to tomato ubiquitin Ubi3 gene.
Three weeks after co-agroinfiltration of two silencing constructs TRV-ACSI+II, or empty vector TRV- control, tomato plants were inoculated with 10,000 second-stage juveniles of Meloidogyne incognita. Three days after nematode inoculation, a portion of the roots was collected from individual plants for gene expression analysis. Silencing efficiency of ACS1A, ACS1B, ACS2, ACS4, ACS5 and ACS6 was evaluated in these root samples by qRT-PCR. Transcripts of ACS1A, ACS4 and ACS5 could not be detected in the control tomato roots. Therefore, results for only ACS1B, ACS2 and ACS6 are presented.
For qRT-PCR, transcripts were amplified from 1 µl of 5x diluted cDNA in a 15 µl reaction using gene-specific primers (Table S2) and iQTM SYBR Green Supermix (Bio-Rad) following the protocol: 94°C for 5 min, cycled 45x [94°C for 30 sec, 58°C for 30 sec, and 72°C for 30 sec], and 72°C for 3 min, followed by generation of a dissociation curve. The generated threshold cycle (Ct) was used to calculate the transcript abundance relative to the housekeeping genes (tomato Ubi3) as described by Ginzinger (2002).
Reference: Ginzinger, D.G. (2002). Gene quantification using real-time quantitative PCR: an emerging technology hits the mainstream. Exp. Hematol. (30): 503-512.
